# Dipeptidyl peptidase-4 (DPP4) inhibitor sitagliptin alleviates liver inflammation of diabetic mice by acting as a ROS scavenger and inhibiting the NFκB pathway

**DOI:** 10.1038/s41420-021-00625-7

**Published:** 2021-09-07

**Authors:** Xin Wang, Jing Ke, Ying-jun Zhu, Bin Cao, Rui-li Yin, Yan Wang, Ling-ling Wei, Li-jie Zhang, Long-yan Yang, Dong Zhao

**Affiliations:** 1 Center for Endocrine Metabolism and Immune Diseases, Beijing Luhe Hospital, Capital Medical University, Beijing, China; 2 Beijing Key Laboratory of Diabetes Research and Care, Beijing, China

**Keywords:** Apoptosis, Cell signalling, Inflammation

## Abstract

As a common chronic metabolic disease, the development of diabetes mellitus (DM) may also be accompanied by liver damage and inflammatory disorders. Sitagliptin is an inhibitor of dipeptidyl peptidase-4 (DPP4, also known as CD26), which is clinically used for DM treatment. However, the mechanism of sitagliptin’s efficiency in liver diseases is largely unknown. In this study, mice suffering from streptozotocin (STZ) exhibit elevated liver DPP4 expression and activity, as well as inflammatory and chronic liver injury phenotype, whereas specifically inhibiting the activity of DPP4 in mouse liver tissues and hepatocytes by sitagliptin contributes to decreased cytokines, oxidative stress, cell apoptosis, and inflammation in STZ-induced diabetic mice. Moreover, sitagliptin reduced TNFα or LPS-induced cellular reactive oxygen species (ROS) level, cell apoptosis, and protein expression in the NFκB signaling pathway in HepG2 cells or primary mouse hepatocytes. Altogether, our study confirms that sitagliptin may protect liver tissue by alleviating ROS production and NFκB signaling activation, providing a putative mechanism for preventing the development of diabetic liver disease.

## Introduction

Diabetes mellitus (DM) is a common chronic metabolic disease, characterized by excessive blood glucose, insulin deficiency, and/or insulin resistance (IR), which can cause coronary artery and cerebrovascular disease, while its damage in microvascular mainly focused on the kidney and retina [1]. Recently, growing studies report the correlation between diabetes with chronic liver disease, such as nonalcoholic fatty liver disease (NAFLD), alcoholic liver disease cirrhosis, and liver cancer, and even colorectal cancer [2, 3]. Meanwhile, diabetes is considered the most common cause of end-stage disease liver cirrhosis and one of the reasons for the high mortality and disability of diabetes patients [4].

Dipeptidyl peptidase-4 (DPP4, also known as CD26) is a serine protease, which is ubiquitously expressed in many cell types and occurs as a soluble form in the circulation system [5, 6]. DPP4 is also highly expressed in the liver and circulation in NAFLD and nonalcoholic steatohepatitis (NASH) patients [7, 8], and there is increasing evidence that DPP4 is highly involved in the development of chronic liver diseases [5, 9]. Sitagliptin, an inhibitor of DPP4, has been clinically used for type 2 DM (T2DM). However, there is also evidence that sitagliptin has been used to rebuild immunological tolerance in type 1 diabetes mellitus (T1DM) and has successfully prevented or even reversed T1DM in non-obese diabetic mice [10, 11]. Meanwhile, clinical evidence shows sitagliptin combined with vitamin D3 effectively achieved the remission of classic clinical manifestations of T1DM in young patients [12]. Early studies have already proved the efficiency and safety of sitagliptin in the treatment of DM complicated with NAFLD and chronic hepatitis caused by NASH or hepatitis C virus [13, 14]. The study of Yoon and colleagues [15] found that sitagliptin destroyed the lipid accumulation in the liver by reducing the expression of DPP4. Otherwise, sitagliptin can effectively alleviate inflammation-related endothelial dysfunction and fibrosis in heart failure model rats by reducing cardiac DPP4 activity, increasing the expression of circulating glucagon-like peptide-1 (GLP-1) and myocardial GLP-1 receptors, and decreasing the level of pro-inflammatory cytokine C–C motif chemokine ligand 2 (CCL2) [16]. By inhibiting the activation of nuclear factor-κB (NFκB), sitagliptin also decreased the expression, extracellular release, and apoptosis of interleukin (IL)-6, IL-1b in lipopolysaccharide (LPS)-induced RINm cells [17]. Early studies showed that sitagliptin induced decreased expression of pro-inflammatory proteins and decreased monocyte migration in apolipoprotein E-deficient mice may be attributed to GLP-1, which reduces monocyte migration and exerts anti-inflammatory effects by decreasing NFκB [18, 19]. As the GLP-1 receptor is not expressed in liver tissue, the downregulation of CCL2 may not be caused by GLP-1 inactivation of NFκB but indirect hypoglycemic effect or direct anti-inflammatory effect of sitagliptin. Otherwise, in Ohnuma et al. [20] study, DPP4 interacts with CARMA1 in T cells, recruits a complex of DPP4-CARMA1-Bcl10-IκB kinase (IKK) to lipid rafts, which further leads to activation of NFκB, and results in signaling events [20].

Hepatic inflammation plays a role in protecting hepatocytes from various hepatotoxic injuries, promoting the reconstruction of homeostasis, and facilitate the repair of tissue damage [21]. However, an overly intense or irreversible inflammatory response is almost always accompanied by a massive loss of hepatocytes, leading to irreversible damage to liver parenchyma [22]. As hepatic stem cell-derived myofibroblasts replace dead hepatocytes, unresolved inflammation can stimulate a fibrosis/cirrhosis response characterized by an irreversible decline in liver function, which significantly increases the risk for hepatic carcinogenesis [23]. Reactive oxygen species (ROS), peroxide anions, and hydrogen peroxide are normal products of the mitochondrial respiratory chain, which mainly produced catalase (CAT) complex I and III, and, as a highly active and diffuse species, ROS is involved in various signal transduction cascades and adaptive stress responses [24]. In physiological conditions, the cytotoxic potential of ROS is controlled by a variety of mitochondrial, cytosolic, and peroxisomal antioxidant systems, including superoxide dismutases (SODs), CATs, peroxiredoxins, thioredoxin, glutathione (GSH), and thiol-containing proteins [24]. However, once ROS levels exceed the buffering capacity on response to multiple stimuli, a series of adaptive responses that are combined with inflammatory responses followed by intracellular damage are activated, which further lead to progressive mitochondrial dysfunction and cell death [25]. According to the latest research, DPP4 inhibitors are known to decrease the ROS level in endothelial cells, kidneys, and the brain [26]. In adipocytes, DPP4 inhibitors are reported to downregulate the expression of NOX4 (nicotinamide adenine dinucleotide phosphate oxidase 4, a major source of ROS) in failing heart [27, 28]. However, there are fewer studies on the effect of DPP4 inhibitors on hepatic oxidative stress and liver disease.

In the present study, we explored the possible mechanisms between sitagliptin and diabetic liver disease. We hypothesized a functional role of sitagliptin in protecting cell apoptosis and inflammation in hepatocytes, as well as delaying the progression of liver disease. Our study provided strong theoretical evidence for the progression management of diabetes patients, with diabetes combined with liver disease.

## Results

### Sitagliptin prevents IKK-NFκB signaling pathway

**Fig. 1:**
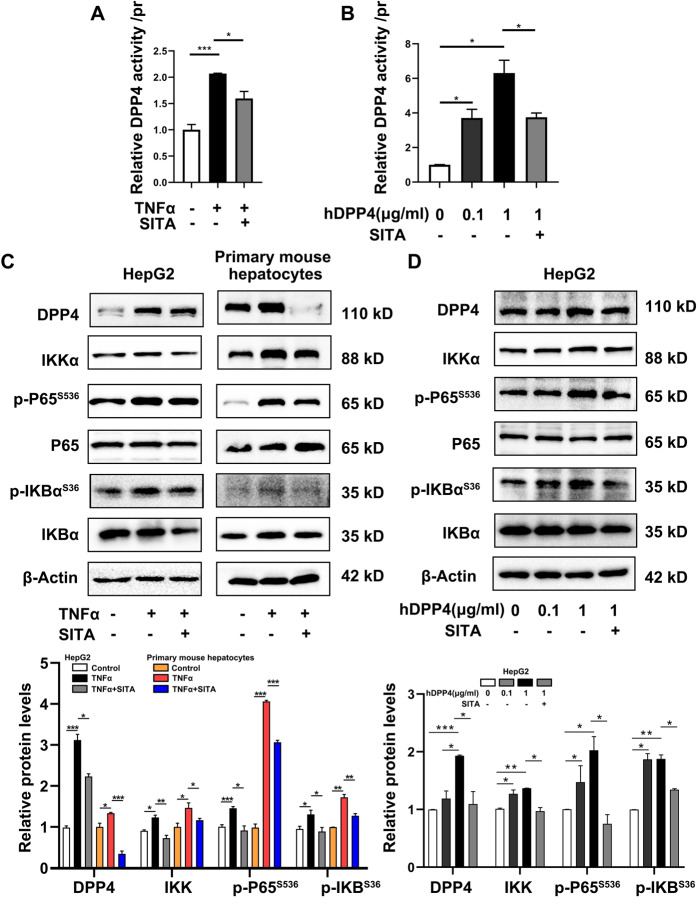
Sitagliptin prevents TNFα-activated NFκB signaling pathway. **A** After 24 h TNFα (20 ng/ml) or TNFα + SITA (100 μM) treatment, DPP4 activity in HepG2 cells were detected. **B** HepG2 cells were co-stimulated recombinant human DPP4 (hDPP4, 0.1 and 1 μg/ml) protein with sitagliptin for 24 h, after that the activity of DPP4 was determined. **C** Western blots and quantitative analysis of the effects of sitagliptin on the protein levels of DPP4, IKKα, p-P65^S536^, P65, IKBα, and p-IKBα^S36^ in TNFα or TNFα + SITA-treated HepG2 cells and primary mouse hepatocytes. **D** Western blottings and quantitative analysis of the effects of different concentrations of hDPP4 on the protein levels of DPP4, IKKα, p-P65^S536^, P65, IKBα, and p-IKBα^S36^ in HepG2 cells. The results are presented as mean ± SEM of three independent experiments; *P < 0.05, **P < 0.01, ***P < 0.001.

We initially mimic the inflammation in vitro with the tumor necrosis factor-α (TNFα)/LPS stimulation in HepG2 and primary mouse hepatocytes. First, the activity of DPP4 was determined and sitagliptin dramatically suppressed the elevation of DPP4 activity stimulated by TNFα/LPS or recombinant human DPP4 (hDPP4) protein (Fig. 1A, B and Supplementary Fig. 1A). In addition, treatment with sitagliptin remarkably inhibited TNFα and LPS increased protein levels of IKKα, p-P65^S536^/P65 ratio, and p-IKBα^S36^/IKBα ratio both in HepG2 cells or primary mouse hepatocytes (Fig. 1C and Supplementary Fig. 1B). Meanwhile, we treated HepG2 cells with different concentrations of hDPP4 and found that the levels of IKKα, p-P65^S536^/P65 ratio, and p-IKBα^S36^/IKBα ratio were significantly enhanced to different degrees, but the activation of IKK-NFκB signaling pathway induced by hDPP4 was remarkably inactivated by sitagliptin (Fig. 1D). These results suggest that sitagliptin may inhibit the IKK-NFκB signaling pathway activated by TNFα, LPS, or hDPP4.

### Sitagliptin prevents cell apoptosis and reduces cellular ROS level

**Fig. 2:**
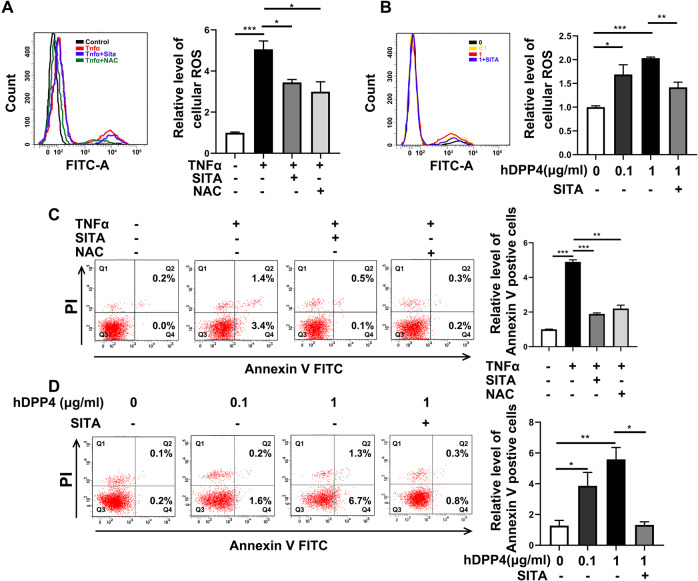
Sitagliptin prevents cell apoptosis and reduces cellular ROS level. **A** Cellular ROS levels of HepG2 cells after TNFα, TNFα + SITA, and TNFα + NAC treatment were detected by flow cytometry. **B** Cellular ROS levels of HepG2 cells after hDPP4 and hDPP4 + SITA treatment were detected by flow cytometry. **C** The apoptosis of HepG2 cells after TNFα, TNFα + SITA, and TNFα + NAC stimulation was detected by flow cytometry. **D** The apoptosis of HepG2 cells after hDPP4 and hDPP4 + SITA stimulation was detected by flow cytometry. The results are presented as mean ± SEM of three independent experiments; *P < 0.05, **P < 0.01, ***P < 0.001.

The development of inflammation is always accompanied by changes in oxidative stress. Although it has been reported that ROS can also affect the activation of NFκB signaling [29]. We then investigated whether sitagliptin ameliorated cellular ROS in TNFα- or LPS-treated HepG2 cells. As shown in Fig. 2A and Supplementary Fig. 1C, sitagliptin significantly reduced the TNFα or LPS-induced cellular ROS level, which is similar to the effect of reductant *N*-acetylcysteine (NAC) treatment (Fig. 2A and Supplementary Fig. 1C). Furthermore, the cellular ROS level was strongly enhanced by different concentrations of hDPP4 treatment in HepG2 cells and dramatically decreased when co-stimulated with sitagliptin (Fig. 2B). In addition, the increased cell apoptosis induced by TNFα or LPS was also remarkably inhibited by the treatment of sitagliptin or NAC compared to the untreated cells (Fig. 2C and Supplementary Fig. 1D). However, the cell apoptosis was significantly increased by different concentrations of hDPP4 treatment in HepG2 cells and decreased when co-stimulated with sitagliptin (Fig. 2D). In addition, NAC treatment may also inhibit the activation of the NFκB signaling pathway in both HepG2 and primary mouse hepatocytes but the expression of DPP4 was not affected (Supplementary Fig. 2). These results may be attributed to the improved cellular oxidative stress. Taken together, these results suggested that sitagliptin may also play a role of ROS scavenger and sitagliptin can prevent the activation of NFκB signaling, which is also affected by the decreased ROS level to some extent.

### Sitagliptin attenuates liver injury and oxidative stress in diabetic mice

**Fig. 3:**
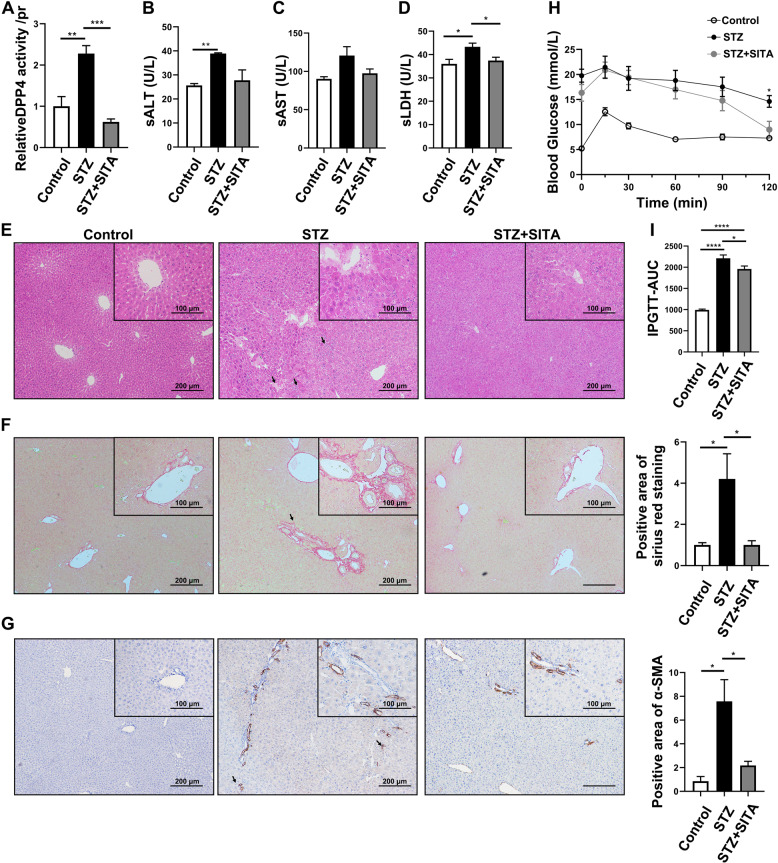
Sitagliptin attenuates STZ-induced liver injury and inflammation. **A** DPP4 activity in liver tissue from STZ-induced diabetic mice with or without 12 weeks of sitagliptin-containing diet treatment. **B**–**D** Levels of sALT, sAST, and sLDH in STZ-induced diabetic mice with or without sitagliptin treatment for 12 weeks. **E** H&E staining of liver tissue (scale bar 100 and 200 μm) in STZ-induced diabetic mice with or without sitagliptin treatment. **F**, **G** Sirius red and α-SMA staining of liver tissue (scale bar 100 and 200 μm) in STZ-induced diabetic mice with or without sitagliptin treatment. **H** After 12 weeks sitagliptin-supplemented diet, mice were fasted overnight and injected with glucose; then, the blood glucose were measured at the specific time points (0, 15, 30, 60, 90, and 120 min). **I** AUC of blood glucose were recorded. *P < 0.05, **P < 0.01, ***P < 0.001, ****P < 0.0001.

We further tested the role of sitagliptin in liver injury. Streptozotocin (STZ)-induced diabetic mice were fed a sitagliptin-containing diet for 12 weeks, to investigate the physiological and pathological changes in liver tissue. As shown in Fig. 3A, DPP4 activity was significantly increased in STZ-diabetic mice, whereas DPP4 activity was significantly inhibited by the treatment with sitagliptin (Fig. 3A). To assess the hepatocellular injury, we detected the level of serum alanine aminotransferase (sALT, Fig. 3B), aspartate aminotransferase (Fig. 3C), and lactic dehydrogenase (sLDH, Fig. 3D). In STZ-diabetic mice, sALT and sLDH were significantly increased and the only sLDH was significantly inhibited by the treatment with sitagliptin. In addition, we showed obvious inflammatory cell infiltration, liver lobule morphology disorder, and the presence of hepatocellular necrosis in diabetic mice, while treatment with sitagliptin significantly improved the mentioned injuries (Fig. 3E). Fibrosis was performed by Sirius red staining and a-SMA (a marker for hepatic stellate cells)-positive staining to observe the collagen deposition (Fig. 3F, G). These results suggested that sitagliptin attenuated STZ-induced liver inflammation and an early stage of fibrosis. By contrast, the glucose response during intraperitoneal glucose tolerance test (IPGTT) significantly improved in the STZ + SITA group (Fig. 3H, *P* < 0.05, T120). After 12 weeks of treatment, the IPGTT-AUC (area under the curve) of the STZ + SITA group was significantly lower than STZ group (Fig. 3I).

To better understand the effect of sitagliptin on STZ-induced oxidative stress, an oxidative stress marker malondialdehyde (MDA), and the level of antioxidant enzymes (GSH, CAT, SOD, and GSH peroxidase (GPx)) in STZ-induced mice with or without sitagliptin treatment were measured. We found that the level of MDA was increased, whereas the antioxidant enzymes were significantly decreased in STZ-induced diabetic mice (Table 1). Treatment with sitagliptin significantly improved STZ-induced hepatic oxidative stress.

**Table 1:** Effects of sitagliptin on oxidative stress markers and antioxidant enzymes in liver tissue of mice.

Groups	MDA (μmol/L/mg prot)	GSH (mg/g prot)	CAT (nmol/min/mg prot)	SOD (U/mg prot)	GPx (pg/ml/mg prot)
Control	2.70 ± 0.72	8.41 ± 1.47	230.5 ± 66.4	0.12 ± 0.07	183.3 ± 34.94
STZ	4.41 ± 0.50^*^	6.59 ± 1.14^**^	156.7 ± 36.11^**^	0.05 ± 0.01^**^	142.0 ± 34.10^**^
STZ + Sita	3.24 ± 0.90^***^	9.73 ± 2.07^***^	223.8 ± 38.63^***^	0.12 ± 0.05^***^	210.9 ± 53.67^***^

Data are presented as mean value ± SEM for groups of six animals each.

*CAT* catalase, *GSH* glutathione, *GPX* glutathione peroxidase, *MDA* malondialdehyde, *SOD* superoxide dismutase.

*p < 0.01 and **p < 0.05 compared with control group.

***p < 0.05 compared with STZ group.

### Sitagliptin inhibits NFκB pathway in diabetic mice during liver inflammation

**Fig. 4:**
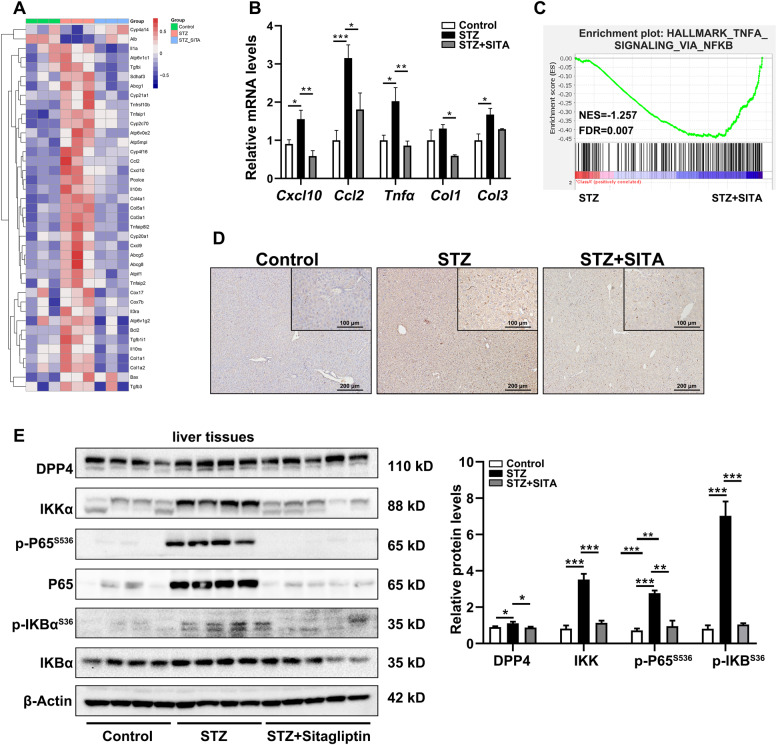
Sitagliptin downregulates NFκB pathway during liver inflammation in STZ-induced diabetic mice. **A** RNA-seq heatmap of the expression of mentioned genes in liver tissues of STZ-induced diabetic mice. **B** Real-time PCR analysis of *Cxcl10*, *Ccl2*, *Tnfα*, *Col1*, and *Col3* levels in diabetic mice with or without sitagliptin treatment. **C** Gene signatures for NFκB activation (HALLMARK_TNFA_SIGNALING_VIA_NFKB) were enriched within STZ group comparing with STZ + SITA group. **D** F4/80 staining of liver tissues (scale bar 100 and 200 μm) in STZ-induced diabetic mice with or without sitagliptin treatment. **E** Western blottings and quantitative analysis of the effects of sitagliptin on the protein levels of DPP4, IKKα, p-P65^S536^, P65, IKBα, and p-IKBα^S36^ in mice liver tissues (*n* = 5 mice per group). The results are presented as mean ± SEM of three independent experiments; *P < 0.05, **P < 0.01, ***P < 0.001.

The liver tissue from control and STZ-induced diabetic mice with or without sitagliptin treatment was performed for RNA sequencing (RNA-seq). These results showed that several cytokines and collagen-related genes were decreased after sitagliptin treatment. In particular, in the downregulated genes with sitagliptin treatment, several vital downstream genes regulated by NFκB, such as *Cxcl10*, *Ccl2*, and *Tnfα* cytokines, are closely related to the process of inflammation (Fig. 4A). As shown in Fig. 4B, a significant decline was confirmed in the mRNA level of *Cxcl10*, *Ccl2*, *Tnfα*, collagen I (*Col1*), and collagen III (*Col3*) in the presence of sitagliptin compared with the untreated STZ-diabetic mice (Fig. 4B). According to the Gene Set Enrichment Analysis (GSEA) assessment, the gene signatures for NFκB were highly enriched in the STZ group (Fig. 4C). Although liver inflammation was also represented by F4/80 (a marker for kuffer cells)-positive staining, sitagliptin significantly alleviated hepatic inflammation in STZ-induced diabetic mice (Fig. 4D). Next, we examined the expression of the related proteins in the IKK-NFκB pathway. Consistently, the increased protein level of IKKa, P65, the ratio of p-P65^S536^/P65, and the ratio of p-IKBα ^S36^/IKBα were significantly attenuated by the treatment with sitagliptin compared with the untreated STZ-diabetic mice (Fig. 4E). These results implied that sitagliptin improved liver inflammation via inhibiting the IKK-NFκB signaling pathway in STZ-diabetic mice.

### Sitagliptin prevents cell apoptosis in diabetic mice

**Fig. 5:**
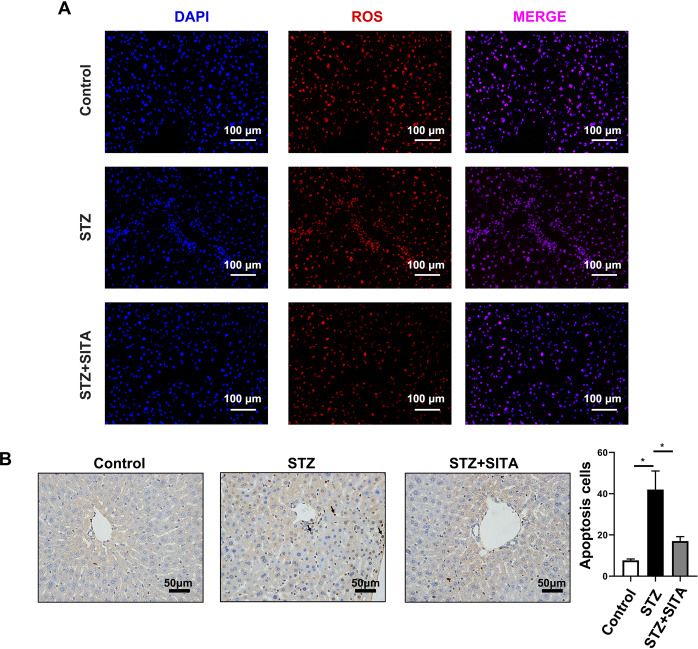
Sitagliptin prevents cell apoptosis in diabetic mice. **A**, **B** TUNEL and ROS staining of liver tissue (scale bar 100 and 50 μm).

It is well known that some systemic metabolic alterations, such as obesity, diabetes, NAFLD, and always persistent chronic irreversible injuries, may lead to an excessive inflammatory response and massive loss of hepatocytes, thereby exacerbating the severity of various liver conditions. As shown in Fig. 5A, sitagliptin significantly decreased the ROS level in hepatocytes (Fig. 5A). Meanwhile, obvious apoptotic terminal deoxynucleotidyl transferase-mediated nick end-labeling (TUNEL)-positive cells were observed in the liver sections from STZ-diabetic mice and sitagliptin effectively reduced cell apoptosis (Fig. 5B).

## Discussion

**Fig. 6:**
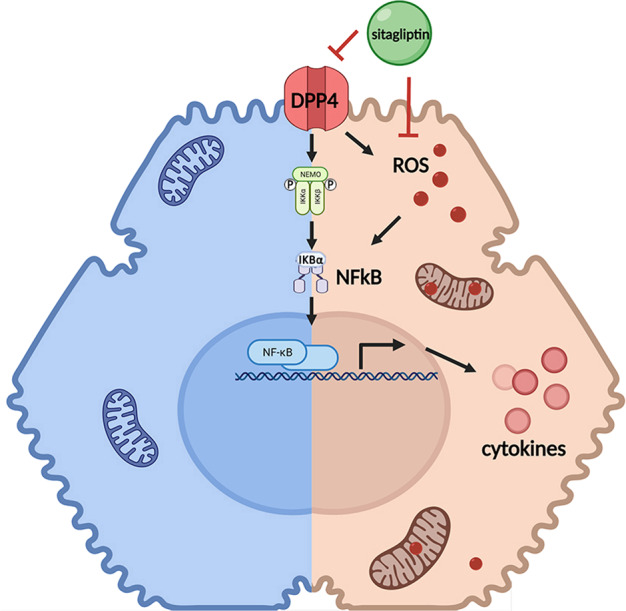
Schematic of the mechanism of sitagliptin in improving liver inflammation. Specifically inhibiting the activity of DPP4 in hepatocytes by sitagliptin contributes to decreased activation of NFκB pathway and oxidative stress, as well as cell apoptosis under diabetic conditions. The ROS cleaning function of sitagliptin promotes the deactivation of NFκB pathway.

Many studies have shown that in addition to pancreatic β-cell damage, STZ can also cause increased serum AFP and liver a-SMA, fibrosis, and necrosis in rats and mice [30, 31]. The present data demonstrate that (1) specifically inhibiting the activity of DPP4 in hepatocytes by sitagliptin contributes to decreased activation of NFκB pathway and oxidative stress, as well as cell apoptosis under diabetic conditions, and (2) the ROS cleaning function of sitagliptin promotes the deactivation of NFκB pathway; moreover, (3) sitagliptin can attenuate STZ-induced chronic liver injury and oxidative stress (Fig. 6).

Liver inflammation, which is usually accompanied in most acute and chronic hepatic disorders, is a complex reaction originating in response to various stress conditions [21]. Dysregulated inflammation is associated with most hepatotoxic injuries, including ischemia/perfusion (IR) injuries, excessive consumption of alcohol, bacterial and virus infections, as well as systemic metabolic conditions such as NAFLD, NASH, diabetes, obesity, and so on [32]. Early studies have suggested that DM might contribute to liver damage by increasing the development of inflammation and fibrosis through adipokine-mediated oxidative stress, such as leptin, adiponectin, IL-6, and TNFα [33, 34]. In fact, effective control of hyperglycemia may have beneficial effects on these patients. In general, diabetes medications focus on the outcome of cardiovascular events, with little attention to liver inflammation and especially the progression of irreversible cirrhosis. It is meaningful to find drugs that improve diabetes and relieve liver damage. Moreover, in transplant patients, especially liver transplant patients, long-term immunosuppressive treatment can cause diabetes, so it is essential for patients to take appropriate diabetes treatment drugs for liver protection [35]. As most drugs, such as oral hypoglycemic agents and insulin, are metabolized by the liver. These drugs may cause hypoglycemia and lactic acidosis [36]. However, little has been reported about the metabolic characteristics of these diabetes drugs. DPP4 inhibitors, such as sitagliptin, vildagliptin, and linagliptin, can increase the secretion of incretin and GLP-1, thereby improving glycemic control without causing hypoglycemia and weight gains [37]. All of these drugs are barely metabolized in the liver but are excreted by the kidneys [38]. In addition, the pharmacodynamics characteristics of these drugs have been evaluated in patients with varying degrees of liver damage and their safety has been evaluated in studies involving large numbers of individuals [39, 40]. However, a randomized placebo-controlled trial reported that 12-week liraglutide or sitagliptin treatment does not reduce hepatic steatosis or fibrosis in T2DM [41]. Besides, an open-label trial comparing sitagliptin and glimepiride found no change in liver fat content after 12 weeks of treatment, but a 15% reduction after 24 weeks [42]. These contradictory results may be due to the differences in duration of treatment, study population, animal models, and evaluation methods. In our study, we found that, as a clinical treatment of DM, sitagliptin also plays a role of ROS scavenger, which can inhibit the activation of NFκB pathway and effectively alleviate chronic liver injury and hepatotoxicity induced by STZ as evidenced by decreased serum ALT, AST, and LDH levels. Meanwhile, sitagliptin can prevent hepatocytes apoptosis and inflammatory response of hepatocytes stimulated by TNFα/LPS.

As a serine protease, DPP4 is capable to cleave various substrates including incretin hormones, growth factors, neuropeptides, and chemokines [43]. Recent research gives evidence that elevated DPP4 expression in the liver promotes NAFLD and IR, which is associated with decreased levels of active GLP-1, but also with the autocrine and paracrine effects of DPP4 on insulin signaling in hepatocytes [44]. DPP4 may also enhance the inflammatory process at the systemic and localized levels by simultaneously stimulating the maturation of T cells, macrophages, and the adhesion of inflammatory extracellular matrix proteins; therefore, the influence of DPP4 inhibitors on these non-catalytic functions of DPP4 cannot be excluded in vivo [45]. However, our in vitro data suggested that sitagliptin is performed as an antioxidant on hepatocytes by reducing the cellular ROS level, and avoiding the activation of a validated signaling pathway, these functions are systemic anti-inflammatory and glucose management independent.

Sitagliptin can also improve the acute liver injury induced by thioacetamide in mice through TLR4/NFκB signaling pathway [46]. Current research on severe acute pancreatitis-related intestinal damage has demonstrated that sitagliptin is capable to reduce the ROS level and alleviate intestinal epithelial cell inflammation, as well as inhibit the NFκB pathway after the activation of Nrf2 [47]. NFκB can promote the inflammatory process by promoting the transcription of various pro-inflammatory genes including cytokines such as TNFα, IL-6, CCL2, and adhesion factors such as VCAM-1 [48]. Consistent with the upregulation of inflammatory molecules, adhesion factors, and CD68 induced by a high-salt diet, the expression of myocardial NFκB was increased as well, whereas sitagliptin treatment could effectively reduce the expression of NFκB [16]. Hepatocyte-secreted DPP4 acts with plasma factor Xa to promote visceral adipose tissue inflammation in obese mice; inflammation and IR of adipose tissue macrophages can also be suppressed by silencing the expression of caveolin-1 (CAV1) or PAR2, which mediated the actions of DPP4 and factor Xa [49]. Previous studies have confirmed that the ligation of CD26 to lipid rafts of T cells via CAV1 recruits a complex consisting of CD26-CARMA1-Bcl10-IKK (Cav1 triggers T-cell activation via CD26 in association with CARMA1) [20], whereas the recruitment of the CARMA1-Bcl10-MALT1 complex activates IKK through a ubiquitin-dependent pathway [50]. In our study, sitagliptin was found to gradually decrease the expression of DPP4 and increase the activation of the NFκB pathway in a dose-dependent manner (Supplementary Fig. 3).

In conclusion, the present study showed that in STZ-induced diabetes model, increased liver DPP4 activity significantly enhanced the tissue oxidative stress and activated NFκB signaling pathway, and further induced chronic liver inflammation. Sitagliptin played as an antioxidant and especially inhibited the activity of DPP4 in hepatocytes, and further reduced cell apoptosis. Based on these results, we concluded that sitagliptin therapy may be effective in improving the progression of liver disorders in patients with diabetes.

## Materials and methods

### Animals and diabetic mouse models

Male, wild-type C57BL/6J mice (8 weeks old) were purchased from GemPharmatech (Nanjing, Jiangsu, China). Mice were adapted to the new environment for 1 week. After that, C57BL/6J mice were randomly assigned to three groups of six mice each. Then, two experimental groups were injected with 60 mg/kg STZ (Sigma-Aldrich, St. Louis, MO, USA, dissolved in 0.1% mol/L citrate buffer [pH 4.5]) for three times every other day, to destroy islet function and generate a diabetic mouse model. As a control, mice were injected with 0.1% mol/L citrate buffer and fed with standard chow diet (Control). After 1 week of the injection, blood glucose ≥ 20 mmol/L was considered as a successful induction of diabetes. Then, mice were fed with standard chow (STZ) or sitagliptin-supplemented diet (40 mg/kg, STZ + SITA) for 12 weeks. All animals were treated humanely and maintained in specific pathogen-free conditions. The experimental protocols were approved by the guideline of the Ethics Committee of the Capital Medical University, Beijing, China.

### Tissue staining and measurement of serum index

After 12 weeks of STZ + SITA, mice were killed by injecting pentobarbital (150 mg/kg body weight) at the termination of the experiments. Liver tissues were fixed in 4% paraformaldehyde and embedded in paraffin for hematoxylin and eosin staining and Sirius red staining.

For a-SMA and F4/80 staining, anti-a-SMA (ab124964, Abcam, Cambridge, UK) and anti-F4/80 (28463-1-AP, Proteintech, Chicago, USA) primary antibodies at 1 : 300 dissolved in 3% bovine serum albumin (BSA)–phosphate-buffered saline at 4 °C overnight. Quantitative results were calculated by Image-Pro Plus software to average the percentage of positive area for five images in each section.

Serum samples were collected for the assay of ALT, AST, and LDH levels with Hitachi 7600-020 clinical analyzer (Tokyo, Japan).

### Intraperitoneal glucose tolerance tests

After 4 weeks of sitagliptin-supplemented diet, mice were fasted overnight, with only water available for glucose tolerance tests. Glucose (2.5 g/kg body weight) was injected intraperitoneally. Blood samples were collected from the mice’s tails and blood glucose was measured at the time points before the injection and after 15, 30, 60, 90, and 120 min. Blood glucose was measured by using ACCU-CHEK^®^ Performa (ROCHE, Mannheim, Germany).

### TUNEL staining

To determine the cell apoptosis in liver tissues, the TUNEL assay was performed according to the manufacturer’s instructions with a commercial kit (Roche, Mannheim, Germany). Quantitative results were calculated by ImageJ by averaging the numbers of TUNEL-positive cells for five fields in each section.

### Cell culture

HepG2 cells were cultured in Dulbecco’s modified Eagle’s medium (DMEM) supplemented with 10% fetal bovine serum (FBS). Primary mouse hepatocytes were isolated from livers of 6–8 weeks male C57BL/6J mice and which were cultured with RPMI 1640 (Gibco, USA) supplemented with 10% FBS and 1% P/S. All cells were sustained at 37 °C with 5% CO_2_ in an incubator.

The inflammatory response was stimulated by two common inducers of cell inflammation—PS (5 μg/ml, dissolved in DMEM, Sigma-Aldrich, St. Louis, MO, USA) or TNFα (20 μM, dissolved in DMEM, Abcam, Cambridge, UK) in the presence or absence of different concentrations (0, 1, 10, 100, and 200 μM) of sitagliptin (dissolved in DMEM, Sigma-Aldrich, St. Louis, MO, USA) for 24 h. In addition, recombinant hDPP4 protein (Abcam, Cambridge, UK) was used to upregulate the expression level of DPP4 in HepG2 cells.

### Measurement of oxidative stress markers and antioxidant enzymes

The level of liver lipid peroxidation product MDA was measurement by using a commercial kit (Applygen Technologies, Beijing, China). The level of liver GSH content and activities of antioxidant defense enzymes including CAT, SOD, and GPx were measured by Elisa kit (Enzyme-linked Biotechnology, Shanghai, China).

### Real-time PCR

According to the manufacturer’s instructions, total RNA from mouse liver and HepG2 cells were extracted with TRIzol (Invitrogen, USA). A HiScript^®^ II 1st Strand cDNA Synthesis Kit was used to get the cDNA sequence (Vazyme Biotech, Nanjing, China). To quantify the expression of mRNA, the SYBR Green Supermix (Bio-Rad, CA, USA) was used. All PCR primers are in Table 2.

**Table 2:** Primers for RT-PCR.

Gene	Forward primer	Reverse primer	Product size (bp)
*mCxcl10*	5′-CCAAGTGCTGCCGTCATTTTC-3′	5′-GGCTCGCAGGGATGATTTCAA-3′	157
*mCcl2*	5′-TAAAAACCTGGATCGGAACCAAA-3′	5′-GCATTAGCTTCAGATTTACGGGT-3′	120
*mTnfa*	5′-CCGGGAGAAGAGGGATAGCTT-3′	5′-TCGGACAGTCACTCACCAAGT-3′	113
*mCol1*	5′-TTCTCCTGGCAAAGACGGAC-3′	5′-CGGCCACCATCTTGAGACTT-3′	198
*mCol3*	5′-AAGGCTGCAAGATGGATGCT-3′	5′-GTGCTTACGTGGGACAGTCA-3′	95

### Western blotting

The expression of DPP4 (ab129060, diluted 1 : 1000, Abcam), IKKα (ab32041, diluted 1 : 5000, Abcam), IKBα (ab32518, diluted 1 : 1000, Abcam), p-IKBα ^S36^ (ab133462, diluted 1 : 1000, Abcam), NFκB1 (14220-1-AP, diluted 1 : 1000, Proteintech), NFκB P65 (P65, ab32536, diluted 1 : 1000, Abcam), NFκB p-P65^S536^ (p-P65^S536^, ab76302, diluted 1 : 1000, Abcam), and GAPDH (10494-1-AP, diluted 1 : 5000, Proteintech) in liver tissues and cell lines were examined by western blotting. Antibody against β-actin (HX1827, diluted 1 : 5000) was purchased from Huaxingbio (Beijing, China). The polyvinylidene difluoride membrane was blocked for 1 h at room temperature by 5% fat-free milk or 5% BSA (dissolved in Tris-buffered saline containing 0.05% Tween-20). After the overnight incubation of the primary antibody at 4 °C, the appropriate horseradish peroxidase-conjugated secondary antibody (Huaxingbio, Beijing, China) was performed. The immunoreactive bands were visualized by ECL chemiluminescent system and exposed with ChemiDoc™ XRS + Imager (Bio-Rad, CA, USA).

### Flow cytometry analysis for apoptosis and ROS

After stimulation, HepG2 cells were collected, a flow-based Annexin V/fluorescein isothiocyanate (FITC) assay (BD) was used to measure the apoptosis of HepG2 cells by following the manufacturer’s instructions. The cells in FITC-positive fraction were considered apoptotic. For ROS, 2 × 10^5^ HepG2 cells were seeded in 12-well plates. After stimulation, dichloro-dihydro-fluorescein diacetate (Applygen Technologies, Beijing, China) were used to incubated cells at 37 °C for 30 min in the dark. Analyses were performed with a FACScan-420 flow cytometry instrument (Becton-Dickinson, USA).

### RNA-seq and data analysis

Liver tissue samples from STZ-induced diabetic mice with or without sitagliptin treatment (12 weeks SITA diet, *n* = 3 per group) were isolated for total RNA by using Trizol (Invitrogen, Carlsbad, CA, USA). The RNA-seq was completed by the BGIseq500 platform (BGI-Shenzhen, China). Data analysis was performed by using R (version 4.0.4) and the “limma” package. |LogFC| > 0.5 and *P*-value < 0.05 were considered significantly different. The GSEA was used to analyze the activation of NFκB pathway in STZ and STZ + SITA groups. The gene set was obtained from the Molecular Signatures Database of the Broad Institute (http://software.broadinstitute.org/gsea/msigdb). False discovery rate (FDR) provides the estimated probability that a gene set with a given normalized enrichment score (NES) represents a false-positive finding; FDR < 0.25 is an accepted cutoff for the identification of biologically significant gene sets.

### DPP4 activity assay

A DPP4 Activity Assay Kit (Abcam, Cambridge, UK) was used for the detection of DPP4 activity by following the manufacturer’s instructions. The results were measured by an Enspire™ Multimode Microplate Reader (PerkinElmer, Waltham, MA, USA).

### Statistical analysis

All results are presented as mean ± SEM. Student’s *t*-test or analysis of variance was used to determine the statistical significance between two or more than two groups. Data were analyzed using GraphPad Prism 8 (GraphPad Software, San Diego, CA, USA). Data are excluded if it deviates from mean with >3 SDs. All *P*-values are two-sided and *P* < 0.05 was considered statistically significant.

## Supplementary information

SUPPLEMENTAL MATERIAL

Figure s1

Figure s2

Figure s3

## Data Availability

All data and material in our study are valid and veritable.

## References

[1] King H, Aubert RE, Herman WH (1998). Global burden of diabetes, 1995-2025: prevalence, numerical estimates, and projections. Diabetes Care.

[2] Zhao Y, Xing H (2017). A different perspective for management of diabetes mellitus: controlling viral liver diseases. J Diabetes Res.

[3] Wild SH, Walker JJ, Morling JR, McAllister DA, Colhoun HM, Farran B (2018). Cardiovascular disease, cancer, and mortality among people with type 2 diabetes and alcoholic or nonalcoholic fatty liver disease hospital admission. Diabetes care.

[4] Harrison SA (2006). Liver disease in patients with diabetes mellitus. J Clin Gastroenterol.

[5] Gorrell MD (2005). Dipeptidyl peptidase IV and related enzymes in cell biology and liver disorders. Clin Sci (Lond).

[6] Lambeir AM, Durinx C, Scharpe S, De Meester I (2003). Dipeptidyl-peptidase IV from bench to bedside: an update on structural properties, functions, and clinical aspects of the enzyme DPP IV. Crit Rev Clin Lab Sci.

[7] Miyazaki M, Kato M, Tanaka K, Tanaka M, Kohjima M, Nakamura K (2012). Increased hepatic expression of dipeptidyl peptidase-4 in non-alcoholic fatty liver disease and its association with insulin resistance and glucose metabolism. Mol Med Rep.

[8] Balaban YH, Korkusuz P, Simsek H, Gokcan H, Gedikoglu G, Pinar A (2007). Dipeptidyl peptidase IV (DDP IV) in NASH patients. Ann Hepatol.

[9] Itou M, Kawaguchi T, Taniguchi E, Sata M (2013). Dipeptidyl peptidase-4: a key player in chronic liver disease. World J Gastroenterol.

[10] Kim SJ, Nian C, Doudet DJ, McIntosh CH (2009). Dipeptidyl peptidase IV inhibition with MK0431 improves islet graft survival in diabetic NOD mice partially via T-cell modulation. Diabetes.

[11] Tian L, Gao J, Hao J, Zhang Y, Yi H, O'Brien TD (2010). Reversal of new-onset diabetes through modulating inflammation and stimulating beta-cell replication in nonobese diabetic mice by a dipeptidyl peptidase IV inhibitor. Endocrinology.

[12] Pinheiro MM, Pinheiro FM, Torres MA. Four-year clinical remission of type 1 diabetes mellitus in two patients treated with sitagliptin and vitamin D3. Endocrinol Diabetes Metab Case Rep. 2016;2016:16-0099. 10.1530/EDM-16-0099 PMC5184778 28035286

[13] Byrne CD (2012). Dorothy Hodgkin Lecture 2012: non-alcoholic fatty liver disease, insulin resistance and ectopic fat: a new problem in diabetes management. Diabet Med.

[14] Hsieh PS, Hsieh YJ (2011). Impact of liver diseases on the development of type 2 diabetes mellitus. World J Gastroenterol.

[15] Cho EY, Ryu JY, Lee H, Hong SH, Park HS, Hong KS (2019). Lecithin nano-liposomal particle as a CRISPR/Cas9 complex delivery system for treating type 2 diabetes. J Nanobiotechnol.

[16] Esposito G, Cappetta D, Russo R, Rivellino A, Ciuffreda LP, Roviezzo F (2017). Sitagliptin reduces inflammation, fibrosis and preserves diastolic function in a rat model of heart failure with preserved ejection fraction. Br J Pharm.

[17] Hu X, Liu S, Liu X, Zhang J, Liang Y, Li Y (2017). DPP-4 (CD26) inhibitor sitagliptin exerts anti-inflammatory effects on rat insulinoma (RINm) cells via suppressing NF-kappaB activation. Endocrine.

[18] Matsubara J, Sugiyama S, Sugamura K, Nakamura T, Fujiwara Y, Akiyama E (2012). A dipeptidyl peptidase-4 inhibitor, des-fluoro-sitagliptin, improves endothelial function and reduces atherosclerotic lesion formation in apolipoprotein E-deficient mice. J Am Coll Cardiol.

[19] Vittone F, Liberman A, Vasic D, Ostertag R, Esser M, Walcher D (2012). Sitagliptin reduces plaque macrophage content and stabilises arteriosclerotic lesions in Apoe (-/-) mice. Diabetologia.

[20] Ohnuma K, Uchiyama M, Yamochi T, Nishibashi K, Hosono O, Takahashi N (2007). Caveolin-1 triggers T-cell activation via CD26 in association with CARMA1. J Biol Chem.

[21] Kubes P, Mehal WZ (2012). Sterile inflammation in the liver. Gastroenterology.

[22] Schattenberg JM, Galle PR, Schuchmann M (2006). Apoptosis in liver disease. Liver Int.

[23] Iwaisako K, Brenner DA, Kisseleva T (2012). What’s new in liver fibrosis? The origin of myofibroblasts in liver fibrosis. J Gastroenterol Hepatol.

[24] Galluzzi L, Kepp O, Trojel-Hansen C, Kroemer G (2012). Mitochondrial control of cellular life, stress, and death. Circ Res.

[25] Jaeschke H (2011). Reactive oxygen and mechanisms of inflammatory liver injury: present concepts. J Gastroenterol Hepatol.

[26] Liu L, Liu J, Tian XY, Wong WT, Lau CW, Xu A (2014). Uncoupling protein-2 mediates DPP-4 inhibitor-induced restoration of endothelial function in hypertension through reducing oxidative stress. Antioxid Redox Signal.

[27] Civantos E, Bosch E, Ramirez E, Zhenyukh O, Egido J, Lorenzo O (2017). Sitagliptin ameliorates oxidative stress in experimental diabetic nephropathy by diminishing the miR-200a/Keap-1/Nrf2 antioxidant pathway. Diabetes Metab Syndr Obes.

[28] Abdelsalam RM, Safar MM (2015). Neuroprotective effects of vildagliptin in rat rotenone Parkinson’s disease model: role of RAGE-NFkappaB and Nrf2-antioxidant signaling pathways. J Neurochem.

[29] Huang Q, Zhan L, Cao H, Li J, Lyu Y, Guo X (2016). Increased mitochondrial fission promotes autophagy and hepatocellular carcinoma cell survival through the ROS-modulated coordinated regulation of the NFKB and TP53 pathways. Autophagy.

[30] Umezu T, Tsuneyama K, Kanekura K, Hayakawa M, Tanahashi T, Kawano M (2020). Comprehensive analysis of liver and blood miRNA in precancerous conditions. Sci Rep.

[31] Uslu S, Alaca N, Kilic KD, Uysal A, Kurtel H (2018). The effects of aerobic exercise frequencies on liver fibrosis, alpha-fetoprotein and cytokeratin 19 in experimental type 2 diabetes-induced rats: an immunohistochemistry study. Biotech Histochem.

[32] Navab M, Gharavi N, Watson AD (2008). Inflammation and metabolic disorders. Curr Opin Clin Nutr Metab Care.

[33] Chang ML, Yang Z, Yang SS. Roles of adipokines in digestive diseases: markers of inflammation, metabolic alteration and disease progression. Int J Mol Sci. 2020;21:8308. 10.3390/ijms21218308 PMC7663948 33167521

[34] Crespo J, Cayón A, Fernández-Gil P, Hernández-Guerra M, Mayorga M, Domínguez-Díez A (2001). Gene expression of tumor necrosis factor alpha and TNF-receptors, p55 and p75, in nonalcoholic steatohepatitis patients. Hepatology.

[35] Grancini V, Resi V, Palmieri E, Pugliese G, Orsi E (2019). Management of diabetes mellitus in patients undergoing liver transplantation. Pharm Res.

[36] García-Compeán D, González-González JA, Lavalle-González FJ, González-Moreno EI, Maldonado-Garza HJ, Villarreal-Pérez JZ (2015). The treatment of diabetes mellitus of patients with chronic liver disease. Ann Hepatol.

[37] Scheen AJ (2012). A review of gliptins in 2011. Expert Opin Pharmacother.

[38] Graefe-Mody U, Rose P, Retlich S, Ring A, Waldhauser L, Cinca R (2012). Pharmacokinetics of linagliptin in subjects with hepatic impairment. Br J Clin Pharm.

[39] Golightly LK, Drayna CC, McDermott MT (2012). Comparative clinical pharmacokinetics of dipeptidyl peptidase-4 inhibitors. Clin Pharmacokinet.

[40] Giorda CB, Nada E, Tartaglino B (2014). Pharmacokinetics, safety, and efficacy of DPP-4 inhibitors and GLP-1 receptor agonists in patients with type 2 diabetes mellitus and renal or hepatic impairment. A systematic review of the literature. Endocrine.

[41] Smits MM, Tonneijck L, Muskiet MH, Kramer MH, Pouwels PJ, Pieters-van den Bos IC (2016). Twelve week liraglutide or sitagliptin does not affect hepatic fat in type 2 diabetes: a randomised placebo-controlled trial. Diabetologia.

[42] Kato H, Nagai Y, Ohta A, Tenjin A, Nakamura Y, Tsukiyama H (2015). Effect of sitagliptin on intrahepatic lipid content and body fat in patients with type 2 diabetes. Diabetes Res Clin Pract.

[43] Rohrborn D, Wronkowitz N, Eckel J (2015). DPP4 in diabetes. Front Immunol.

[44] Baumeier C, Schlüter L, Saussenthaler S, Laeger T, Rödiger M, Alaze SA (2017). Elevated hepatic DPP4 activity promotes insulin resistance and non-alcoholic fatty liver disease. Mol Metab.

[45] Zhong J, Rajagopalan S (2015). Dipeptidyl peptidase-4 regulation of SDF-1/CXCR4 axis: implications for cardiovascular disease. Front Immunol.

[46] El-Kashef DH, Serrya MS (2019). Sitagliptin ameliorates thioacetamide-induced acute liver injury via modulating TLR4/NF-KB signaling pathway in mice. Life Sci.

[47] Zhou X, Wang W, Wang C, Zheng C, Xu X, Ni X (2019). DPP4 Inhibitor Attenuates Severe Acute Pancreatitis-Associated Intestinal Inflammation via Nrf2 Signaling. Oxid Med Cell Longev.

[48] Adams V, Späte U, Kränkel N, Schulze PC, Linke A, Schuler G (2003). Nuclear factor-kappa B activation in skeletal muscle of patients with chronic heart failure: correlation with the expression of inducible nitric oxide synthase. Eur J Cardiovasc Prev Rehabil.

[49] Ghorpade DS, Ozcan L, Zheng Z, Nicoloro SM, Shen Y, Chen E (2018). Hepatocyte-secreted DPP4 in obesity promotes adipose inflammation and insulin resistance. Nature.

[50] Sun L, Deng L, Ea CK, Xia ZP, Chen ZJ (2004). The TRAF6 ubiquitin ligase and TAK1 kinase mediate IKK activation by BCL10 and MALT1 in T lymphocytes. Mol Cell.

