# Supplementary material for: Dipeptidyl peptidase-4 (DPP4) inhibitor sitagliptin alleviates liver inflammation of diabetic mice by acting as a ROS scavenger and inhibiting the NFκB pathway
Source: Cell Death Discov. 2021 Sep 7;7:236. doi: 10.1038/s41420-021-00625-7 (PMC8423797; doi:10.1038/s41420-021-00625-7)
Supplement: Supplementary file 1 — SUPPLEMENTAL MATERIAL [file 41420_2021_625_MOESM1_ESM.docx]

**Figure legends**

Suppl. Figure 1. Sitagliptin prevented LPS induced cell apoptosis and the activation of NFκB signaling pathway.

A. After 24 h LPS (5 μg/ml) or LPS +SITA (100 μM) treatment, DPP4 activity in HepG2 cell were detected. B. Western blots and quantitative analysis of the effects of sitagliptin on the protein levels of DPP4, IKKα, p-P65^S536^, P65, IKBα, and p-IKBα^S36^ in LPS or LPS+SITA treated HepG2 cell and primary mouse hepatocytes. C. Effects of sitagliptin or NAC (2 h pretreatment before LPS stimulation) on cellular ROS production in HepG2 cells. D. After 24 h LPS, LPS+SITA, and LPS+NAC treatment, cell apoptosis of HepG2 was measured by flow cytometry. The results are presented as mean ± SEM of three independent experiments. **P* < 0.05, ***P* < 0.01, ****P* < 0.001.

Suppl. Figure 2. Reducing the cellular ROS level inhibited the activation of NFκB signaling pathway.

Western blots and quantitative analysis of the effects of NAC on the protein levels of DPP4, IKKα, p-P65^S536^, P65, IKBα, and p-IKBα^S36^ in TNFα or TNFα+SITA treated HepG2 cell and primary mouse hepatocytes. The results are presented as mean ± SEM of three independent experiments, **P* < 0.05, ***P* < 0.01.

Suppl. Figure 3. Sitagliptin dose-dependently inhibited the activation of NFκB signaling pathway.

HepG2 cells were treated with various doses of sitagliptin (0, 1, 10, 100, 200 μM) for 24 h accompanied by TNFα or LPS, and the protein levels of DPP4, IKKα, p-P65^S536^, P65, NFKB1, IKBα, and p-IKBα^S36^ were measured by western blots.
