# Supplementary figures and images for: Dipeptidyl peptidase-4 (DPP4) inhibitor sitagliptin alleviates liver inflammation of diabetic mice by acting as a ROS scavenger and inhibiting the NFκB pathway
Source: Cell Death Discov. 2021 Sep 7;7:236. doi: 10.1038/s41420-021-00625-7 (PMC8423797; doi:10.1038/s41420-021-00625-7)

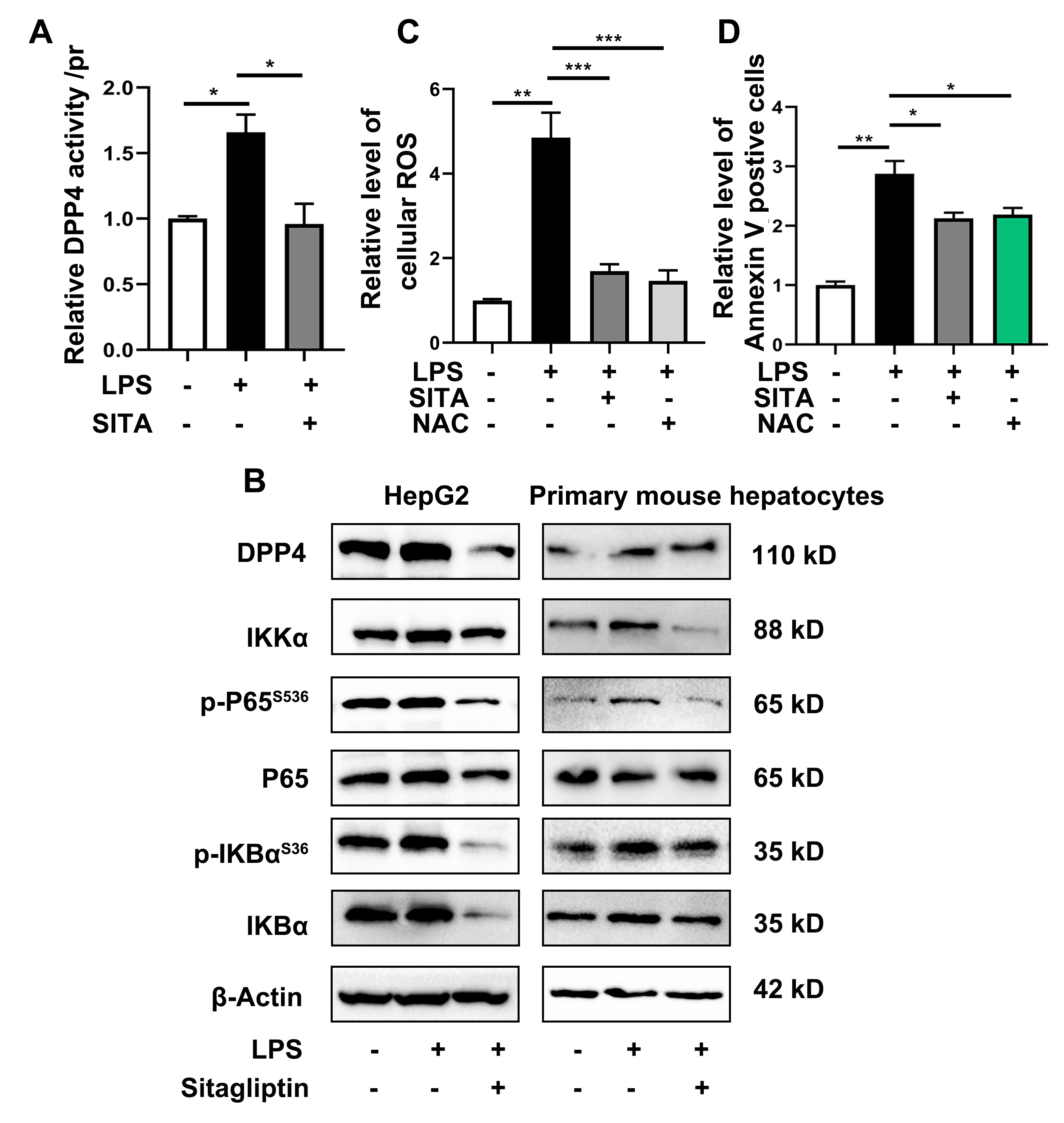

Supplement: Supplementary file 2 — Figure s1 [file 41420_2021_625_MOESM2_ESM.jpg]

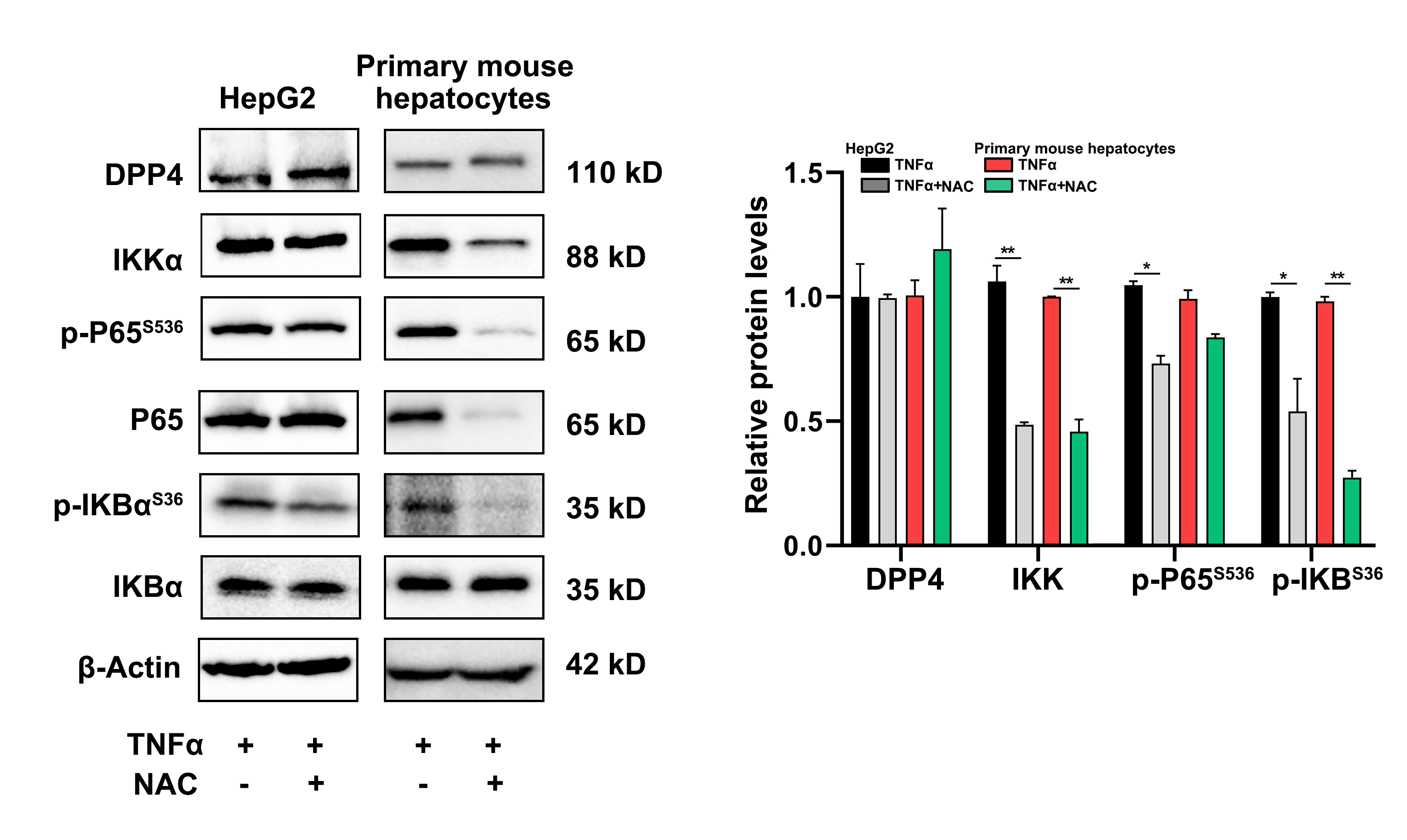

Supplement: Supplementary file 3 — Figure s2 [file 41420_2021_625_MOESM3_ESM.jpg]

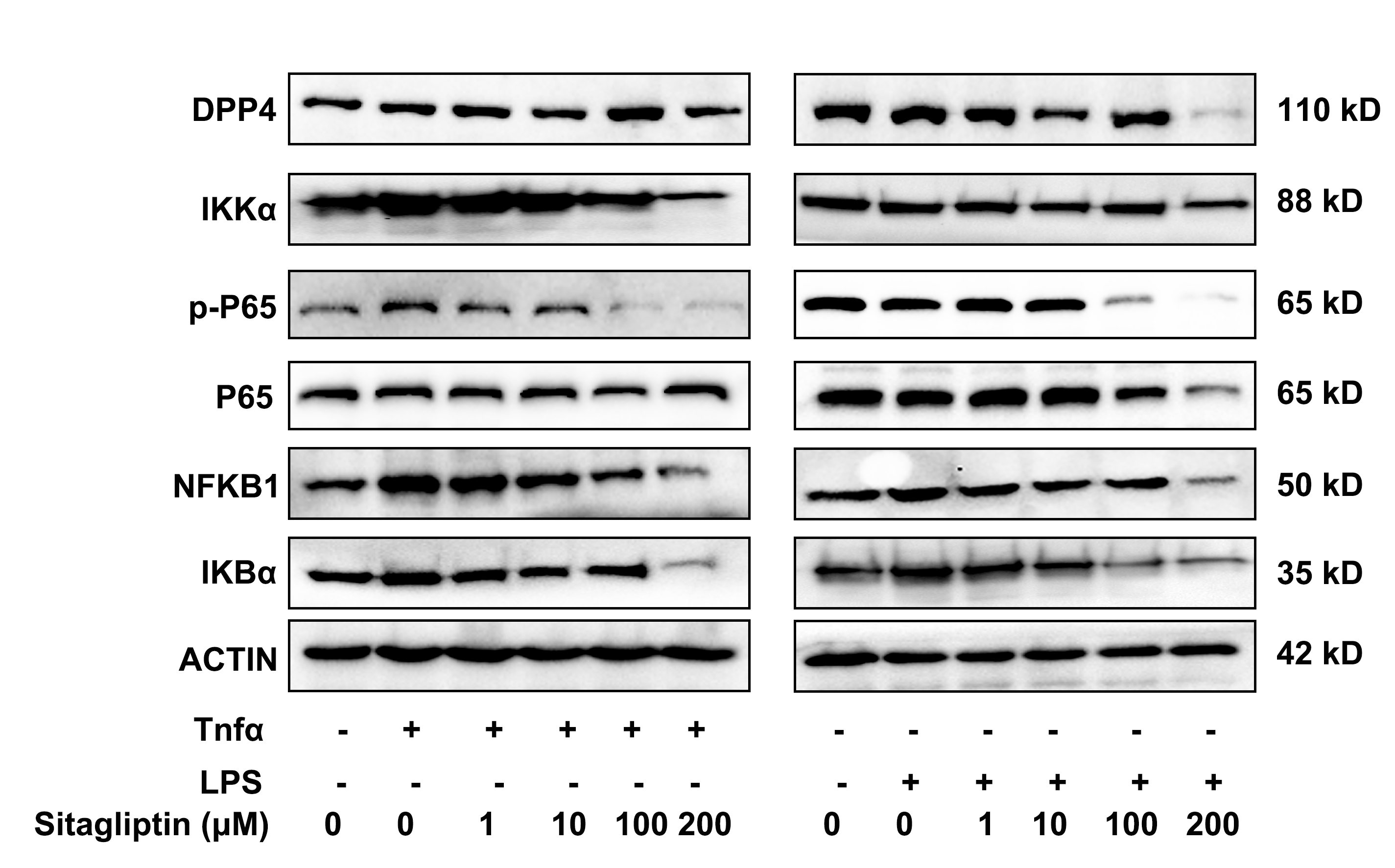

Supplement: Supplementary file 4 — Figure s3 [file 41420_2021_625_MOESM4_ESM.jpg]
